# The Effect of Oral Care Foams and a Spray on Salivary pH Changes after Exposure to Acidic Beverages in Young Adults

**DOI:** 10.3390/dj12040093

**Published:** 2024-04-03

**Authors:** Maria Polyakova, Anna Egiazaryan, Vladlena Doroshina, Alexandr Zaytsev, Alexey Malashin, Ksenia Babina, Nina Novozhilova

**Affiliations:** 1 Department of Therapeutic Dentistry, I.M. Sechenov First Moscow State Medical University (Sechenov University), 119991 Moscow, Russia; polyakova_m_a_1@staff.sechenov.ru (M.P.); anylechka@mail.ru (A.E.); doroshina_v_yu@staff.sechenov.ru (V.D.); novozhilova_n_e@staff.sechenov.ru (N.N.); 2 Institute of Linguistics and Intercultural Communication, I.M. Sechenov First Moscow State Medical University (Sechenov University), 119991 Moscow, Russia; zaytsev_a_b@staff.sechenov.ru; 3 Independent Researcher, 123298 Moscow, Russia; alexey@malash.in

**Keywords:** carbonated beverages, salivary pH, tooth erosion

## Abstract

Soft drinks may have a deleterious effect on dental health due to a high titratable acidity and a low pH that could be sufficient to induce tooth demineralization. The use of oral care products immediately after acidic challenge may diminish the erosive potential of soft drinks. We assessed the effect of oral care foams and a spray on salivary pH changes after exposure to Coca-Cola^®^ in young adults. Thirty-three consenting eligible patients were recruited in this double-blind, randomized, crossover study performed in six visits. Baseline examination included unstimulated salivary flow rate, stimulated salivary buffer capacity, and the simplified oral hygiene index (OHI-S) assessment. Salivary pH and time for pH recovery were registered after exposure to Coca-Cola^®^ alone or that followed by the application of each of the studied products (an oral foam containing hydroxyapatite and probiotics, an oral foam containing amino fluoride, an alkaline oral spray, and tap water). Thirty-two patients completed the entire study protocol and were included in the final analysis. The mean minimum salivary pH and the mean oral clearance rate after rinsing with Coca-Cola^®^ were 6.3 and 27 min, respectively. Further rinsing with any one of the tested solutions, including tap water, resulted in a significant improvement in these parameters. When the pH curves were plotted, the oral care products demonstrated a lower area under the curve that differed significantly from the area under the curve for Coca-Cola^®^; tap water did not differ significantly from Coca-Cola^®^ and oral care products. Minimum salivary pH correlated positively with salivary buffer capacity and salivation rate, while salivary clearance correlated with OHI-S plaque scores. In conclusion, the effect of oral care foams and a spray on minimum salivary pH and salivary clearance after exposure to Coca-Cola^®^ did not differ significantly among the tested products and tap water. Trial registration NCT06148662. Funding: none.

## 1. Introduction

Soft drinks such as colas or orange sodas are extremely popular across the world [1]. Over the past decades, consumption of these beverages has substantially increased in most parts of the world [2,3,4,5]. The prevalence of soft drink consumption is especially high among young people [6,7,8,9,10]. In addition, a rise in the use of carbonated beverages was observed during the COVID-19 pandemic [11].

It has been found that soft drinks may have a deleterious effect on general [12,13,14] and dental [15,16] health. A recent review [16] reported strong evidence for an association between carbonated drink consumption and oral health problems, including increased risks of periodontitis [17], dental caries, and erosion [18,19]. Dental erosion is the irreversible loss of hard tooth tissues caused by direct contact with intrinsic or extrinsic acids without bacterial involvement [20,21]. The prevalence of erosive tooth wear was shown to be especially high in adolescents and young adults [22,23,24,25]. A meta-analysis by Salas et al. (2015) confirmed that frequent consumption of carbonated drinks increased the odds of tooth erosion [24]; therefore, the high prevalence of dental erosion in young adults may be partially explained by the high frequency of soft drink consumption [26].

The deleterious effects of soft drinks on hard tooth tissues can be related to two main factors. The first factor is organic acids produced by plaque micro-organisms when metabolising fermentable carbohydrates from the soft drinks [27,28]. The second factor is the low pH and titratable acidity of the drinks themselves [27]. Most soft drinks, excluding bottled waters, have a pH that ranges from 2.5 to 3.5 [29], as they are carbonated and commonly contain acids added to enhance flavour (phosphoric acid, malic acid, citric acid, etc.) [30,31]. As a consequence, they show a high titratable acidity and a low pH that could be sufficient to induce tooth demineralization [32]. These acids, if not neutralized, are capable of dissolving the enamel surface [27].

Saliva plays a key role in maintaining the integrity of the enamel due to its buffering ability that controls the demineralization/remineralization equilibrium at the enamel surface [33]. A recent systematic review has shown that of all potential risk factors associated with saliva, whole saliva pH showed the strongest negative association with tooth wear [34]. The role of salivary buffer systems is to maintain the salivary pH at a relatively constant level (i.e., 6.5–7) by neutralizing acids from food and drinks and those produced by bacteria, thus decreasing the tooth demineralization rate [33].

A number of studies have assessed salivary pH dynamics after consumption of various soft drinks. The existing studies vary in experimental design, namely in their saliva collection conditions, type of exposure to soft drinks, and types of soft drinks assessed; therefore, their results are difficult to compare. In general, they found that soft drink consumption was associated with a salivary pH drop of different degrees [35,36,37,38,39,40], followed by a gradual recovery to baseline pH levels due to salivary clearance and buffering [36,38]. However, these protective mechanisms may not be sufficient in cases of a decreased salivation rate [35] or frequent exposure to acidic food or beverages [36]. It has been found that daily consumption of more than four acidic units is strongly associated with enamel erosion development [41,42,43].

Several approaches have been proposed to diminish the deleterious effects of soft drinks on hard tooth tissues. Some authors have proposed increasing salivation by chewing cheese or chewing gum to counteract the erosive potential of acid beverages [44,45,46]. Others have tested modifications of various soft drinks with low concentrations of calcium or a combination of calcium, phosphate, and fluoride. It has been shown that the addition of calcium and phosphate to the experimental drinks considerably decreased their erosive potential [47,48,49]. Another approach is to use oral care products immediately after acidic exposure. Regarding anti-erosive and remineralizing potential, the most promising results have been reported for fluoride- and hydroxyapatite-containing products [20,50,51,52,53,54,55,56]. Alkaline oral care products may also be of interest due to their ability to increase salivary pH [57,58,59]. Several studies have assessed the neutralizing effect of mouthwashes containing these ingredients and have concluded that they might potentially reduce tooth erosion caused by acid exposure [57,60,61]. However, the literature is lacking regarding the anti-erosive potential of oral care products with different compositions and forms. Moreover, with the development of new oral care products with various active ingredients and characteristics, it is important to continuously update evaluations of their effects. Oral care foams and sprays may be considered an alternative to mouthwashes in cases where the use of the latter is inconvenient, as only few mouthwashes are available in portable size packages. It was found that oral foams can increase salivation [62]. To the best of our knowledge, there have been no previous studies regarding the neutralizing effect of oral foams or sprays with different compositions. Therefore, we aimed to assess the effect of three oral care products (an oral foam with fluoride, an oral foam with Zn-hydroxyapatite and probiotics, and an alkaline spray) on salivary pH changes after exposure to Coca-Cola^®^ in young adults.

## 2. Materials and Methods

### 2.1. Ethical Approval

The Ethics Committee of Sechenov University approved the study (Protocol no. 04-23, 2 March 2023). The study registration number on clinicaltrials.gov is NCT06148662.

### 2.2. Study Design

This double-blind, randomized, crossover study was conducted from November 2023 to December 2023 at the Therapeutic Dentistry Department, Sechenov University, Moscow, Russia. The study included six visits. After enrolment, dental examination was performed using the following indices: the Decayed, Missing, and Filled Teeth index (DMFT), the Simplified Oral Hygiene Index (OHI-S), and the Bleeding index (BI) [63,64,65]. Stimulated salivary buffer capacity, unstimulated salivary flow rate, and baseline salivary pH were measured. Salivary pH changes were registered after exposure to Coca-Cola^®^ alone or followed by the application of each of the following products: an oral foam containing zinc hydroxyapatite and probiotics (BF), an oral foam containing amino fluoride (WF), an oral spray with alkaline thermal water (BS), and tap water as a control. The pH and titratable acidity of the solutions was measured using a digital pH meter (MILWAUKEE PH56 PRO, Rocky Mount, NC, USA). To assess the titratable acidity, 50 mL of each solution was titrated by adding 0.1 mL aliquots of NaOH (0.1 M) until the pH of 7.0 and the amount of NaOH (mmol) needed for neutralization was calculated. All measurements were repeated 3 times and the results were averaged. Table 1 shows the active ingredients and pH and titratable acidity values of the products used in the study.

### 2.3. Sampling Criteria

Thirty-two young adults of both genders aged 18–44 years were enrolled by two study authors (MP and AE). Participants were free to withdraw from the study at any point.

#### 2.3.1. Inclusion Criteria

The inclusion criteria were males and females aged 18–44 years; all participants signed informed consent forms for participation in the study and publication of the data for research and education purposes.

#### 2.3.2. Exclusion Criteria

Patients with systemic or local conditions that may affect the study results or in which the exposure to Coca-Cola is contraindicated were not enrolled in the study. Systemic conditions included diseases (e.g., diabetes mellitus, Sjögren syndrome, chronic kidney disease, arterial hypertension, etc.) and/or intake of medications (e.g., of antihypertensive, anti-acne, anticholinergic, and antiseizure medications, antidepressants, antihistamines, etc.) that may affect salivary parameters. Pregnant and breastfeeding patients were also not included due to physiologic hormonal changes that are known to affect salivary parameters. Local conditions included oral mucosa pathology as it may affect salivation rate; moreover, the presence of any pathological lesions on the oral mucosa makes the exposure to acidic beverage and any new oral care product unacceptable. Orthodontic appliance treatment was an exclusion criterion as it may alter salivation and affect oral hygiene. Finally, dental bleaching makes the exposure to acidic and coloured beverage undesirable, thus it was also considered an exclusion criterion.

#### 2.3.3. Elimination Criteria

The patients were eliminated from the study in case of withdrawal of consent, detection of an allergic reaction to any components of the of the products used in the study; prescription of medicaments after the enrolment that may compromise the protocol, and non-compliance with the study protocol (failure to attend any of the six visits).

### 2.4. Randomization

The allocation concealment was performed using a randomized sequence of oral care products for each patient applied by an operator who did not participate in the study. The oral care products were in unlabelled non-transparent bottles. Neither patients nor researchers were aware of the type of the oral care product used.

### 2.5. Interventions and Outcomes

At the baseline visit, the following indices were assessed: DMFT, OHI-S, and BI (as described elsewhere [63,64,65]). Demographic characteristics, medical and medication history were registered. Two trained and calibrated researchers, MP and AE (intra- and inter-examiner Kappa agreement of at least 94%), performed all clinical examinations.

Unstimulated salivary flow rate, baseline salivary pH, and stimulated saliva buffer capacity were assessed. Unstimulated saliva samples were collected between 10 and 11 am. Participants abstained from food, drink, smoking, or oral hygiene procedures for at least 1.5 h before the procedure. Participants sat comfortably, did not swallow saliva, and expectorated it into a graduated tube every two minutes to assess salivary flow rate. The salivation rate (mL/min) was calculated as the saliva volume collected within the 10 min period divided by 10 min. Salivation rate was classified as follows: low (0.046–0.264 mL/min), medium (0.265–0.451 mL/min), or high (0.451–1.850 mL/min) [66]. Salivary pH was measured immediately after the collection using a pH meter (MILWAUKEE PH56 PRO, Rocky Mount, NC, USA).

To assess salivary buffer capacity, 0.5 mL of stimulated saliva was mixed with 1.5 mL of HCl (5 mM) in a plastic vial and left it open for 5 min for CO_2_ release. Then, the pH of the mixture was measured. Both tests (salivation rate and buffer capacity) for each participant were repeated thrice and their results were averaged.

The second visit was scheduled after 1 week. Participants’ salivary baseline pH levels were measured as described above. The participants held 20 mL of Coca-Cola^®^ beverage in the mouth for 30 s. After that, the Coca-Cola^®^ was eliminated (either spat out or swallowed). Then, the participants spat saliva into a sterile glass tube and salivary pH was measured within 1 min (time required to collect enough saliva and perform the measurement) after exposure to the beverage, 2 min after the exposure, and then every 5 min until the pH reached the baseline levels. The time required for pH recovery was registered.

The following visits were performed at weekly intervals. Participants’ salivary baseline pH levels were measured as described above. The participants held 20 mL of Coca-Cola^®^ beverage in the mouth for 30 s. After that Coca-Cola^®^ was eliminated (either spat out or swallowed). Then the participants spat saliva into a sterile glass tube and salivary pH was measured within 1 min (time required to collect enough saliva and perform the measurement). Next, one of the tested oral care products was used according to the instructions of the manufacturer and pH levels were measured immediately after application (2 min measurement) and then every 5 min until the pH reaching baseline levels. The time required for pH recovery was registered.

The tested products were used as follows:

1. WF and BF were squeezed (2 pumps of the foams) into the mouth, swished for 30 s, and spat out without rinsing.
2. BS was sprayed in the mouth (2 sprays) and left for 30 s; could be swallowed.
3. A sip of tap water was swished in the mouth for 30 s and spat out.

Primary outcome measures included minimal pH level and salivary clearance time after exposure to Coca-Cola^®^ alone or followed by rinsing with the studied solutions. Salivary clearance time was the time required for the salivary pH to reach the baseline level. Area under the curve was calculated as the area between the baseline pH level and the part of the curve where the pH remained below the baseline pH level [67].

### 2.6. Statistical Analysis

The sample size was calculated for a Wilcoxon matched-pairs signed-rank test (effect size was assumed to be medium, i.e., 0.5). G*Power (version 3.1.9.6, Institute for Experimental Psychology, Dusseldorf, Germany) was used for sample size calculation: the power was set at 80% and alpha level was set as 0.05. The target sample size was 33 participants in each group (sample size calculations yielded 28 participants and with 15% were added to compensate for potential dropout).

Means, standard deviations, and medians and interquartile ranges were used to present continuous variables. The Friedman test was used to assess statistical differences among the tested solutions, A pairwise signed ranks post hoc test with a Benjamini–Hochberg procedure was used to adjust for multiple comparisons. Kendall’s W was used to calculate the effect size for each variable. Spearman’s correlation coefficient was used to accomplish correlation analysis.

### 2.7. Data Management

The data were entered in the MS Excel database (Excel for Mac version 16.79.1 (23111614), Microsoft Corp., Mountain View, CA, USA) and converted to the CSV file format. Pseudonymized data were analysed in R (version 4.2.3 (15 March 2023), R Development Core Team, Columbia university, New York, NY, USA) using “rstatix” and “ellipse” packages in RStudio version 2023.03.0+386.

## 3. Results

**Figure 1:**
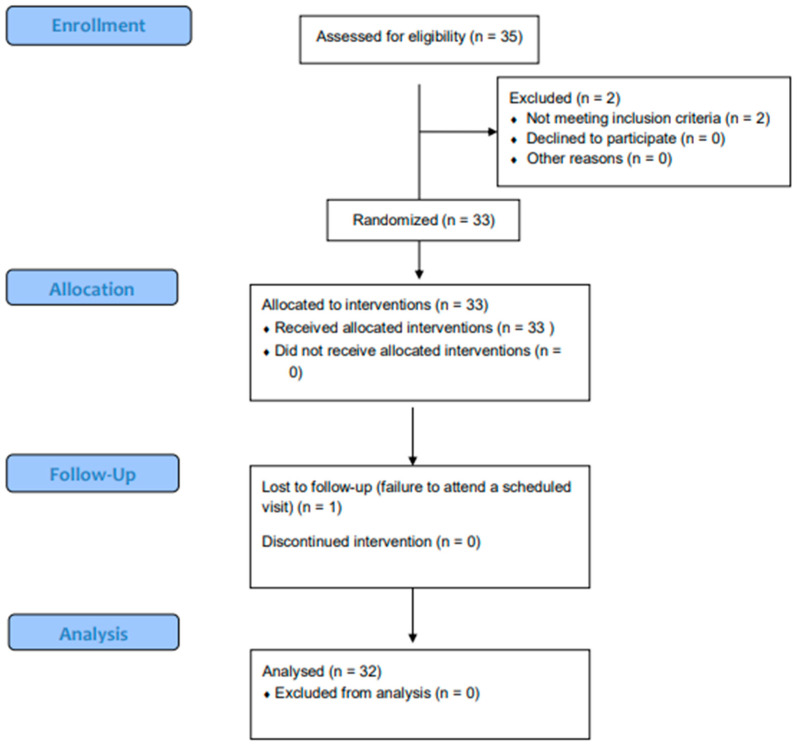
Participant flow chart.

Thirty-five patients aged 18 to 44 years were assessed for eligibility. Of them, two patients were ineligible due to meeting the exclusion criteria. A total of 33 patients were enrolled in the study (24 females and 9 males). One participant was lost to follow up (*n* = 1). A total of 32 participants (mean age = 23.8 ± 6.1 years) were included in the final analysis (Table 2, Figure 1).

Table 3 shows the oral health status of the participants. The mean DMFT value in the study population was 7.8 teeth (low caries intensity), with the FT component being predominant (mean value 6.2). Forty-nine percent of the study participants presented with fair oral hygiene. The mean BI value was 0.14 points.

The salivary parameters profile of the study participants showed that the majority of participants had a high salivation rate (58.9%) and high buffer capacity (10.7%) Table 4.

**Figure 2:**
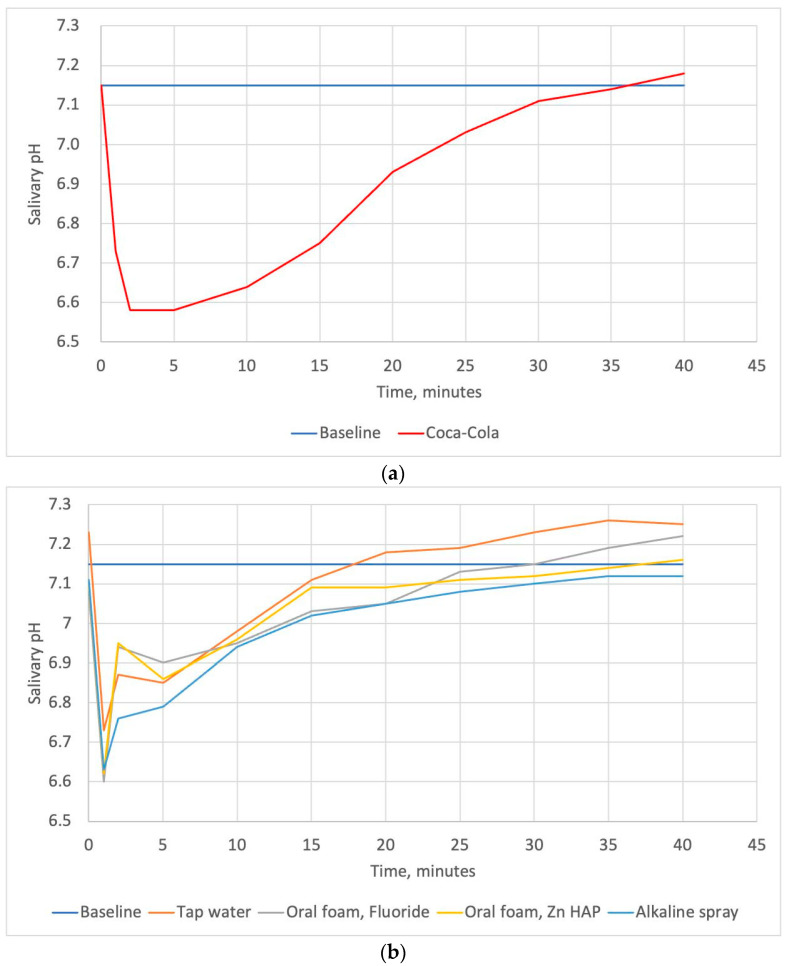
The pH–time curves plotted using mean salivary pH readings for all subjects after exposure to Coca-Cola^®^ (**a**) and the pH–time curves plotted using mean salivary pH readings for all subjects after exposure to Coca-Cola^®^ followed by rinsing with oral care foams, a spray, and tap water (**b**); “Baseline” line represents the mean salivary pH level before exposure to the beverage.

Figure 2 shows the pH curves plotted using mean pH readings for all subjects after exposure to Coca-Cola^®^ and rinsing with different liquids over time. The mean salivary pH of the subjects at baseline was 7.18 ± 0.22. The maximum drop in pH was observed within the first 2 min. Then, a gradual recovery of the salivary pH was observed in all groups. The quantitative description of the minimum pH, and time required for salivary clearance, as well as the areas under the pH curves are also presented in Table 5.

Table 5 shows the mean minimum salivary pH and the mean oral clearance rate of Coca-Cola^®^ after rinsing with tap water or tested oral care products. The mean minimum salivary pH after exposure to Coca-Cola^®^ was 6.3. This parameter increased significantly after rinsing with any one of the tested solutions, with no significant differences among them. The mean oral clearance rate of Coca-Cola^®^ without rinsing was found to be 27 min. Rinsing with any solutions resulted in a significant decrease in salivary clearance time. We found no significant differences in this parameter among the rinsing solutions. However, the salivary clearance time after the use of BS (alkaline spray) tended to be the lowest and comprised 16 min. The areas under pH curves plotted for the Zn-hydroxyapatite-containing foam, fluoride-containing foam, and alkaline spray differed significantly from that for Coca-Cola^®^ (*p* = 0.002, *p* = 0.004, and 0.036, respectively). Tap water did not differ significantly from Coca-Cola^®^ and oral care products in this parameter.

**Figure 3:**
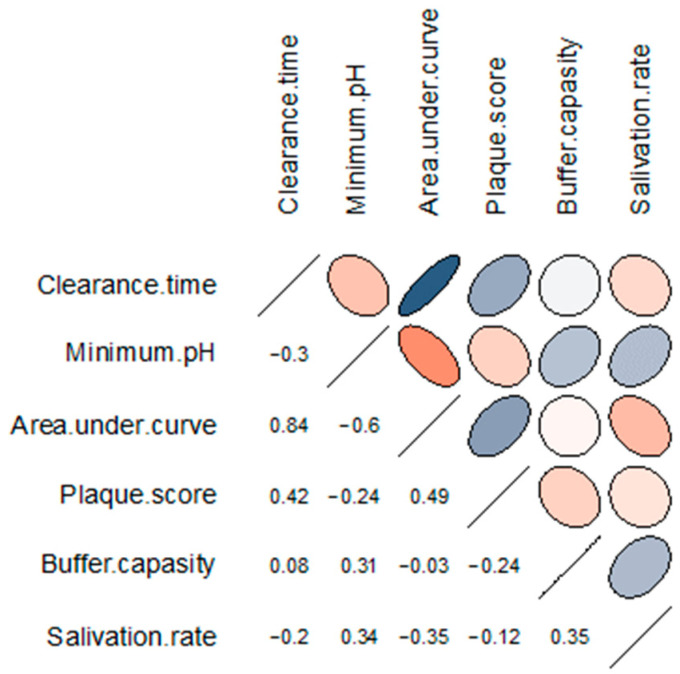
Correlation matrix of the salivary parameters and oral hygiene index after exposure to Coca-Cola^®^. The rows and columns represent the correlating variables. In the upper triangular portion of the matrix, positive correlations are displayed in blue and negative correlations in red; the intensity of the colour is proportional to the strength of the correlation. The lower triangular portion represents the values of Spearman’s correlation coefficient between the pairs of variables.

Figure 3 denotes the results of the correlation matrix of oral hygiene and salivary parameters after exposure to Coca-Cola^®^. We found a moderate but not quite statistically significant positive correlation between the minimum salivary pH and unstimulated salivation rate (r = 0.34, *p* = 0.05782) and salivary buffer capacity (r = 0.31, *p* = 0.08585). Also, a moderate positive correlation was detected between salivary buffer capacity and unstimulated salivation rate (r = 0.35, *p* = 0.04765). Salivary clearance values moderately correlated with OHI-S plaque scores (r = 0.42, *p* = 0.01555). The area under the pH curve correlated positively with salivary clearance time (r = 0.84, *p* < 0.0001) and OHI-S plaque scores (r = 0.49, *p* = 0.00467) and negatively with minimum salivary pH (r = −0.60, *p* = 0.000327) and unstimulated salivary flow rate (r = −0.35, *p* = 0.04664).

## 4. Discussion

In our study, we assessed the effect of two oral care foams and a spray on salivary pH changes after exposure to Coca-Cola^®^ in young adults. We found that rinsing with any one of the tested solutions, including tap water, resulted in a significant decrease in salivary clearance time and in a significant increase in the minimum pH. When the pH curves were plotted, the oral care products demonstrated a lower area under the curve that differed significantly from the area under the curve for Coca-Cola^®^; tap water did not differ significantly from Coca-Cola^®^ and oral care products in this parameter. Salivary clearance values and the area under the pH curve moderately correlated with OHI-S plaque scores; the area under the pH curve negatively correlated with unstimulated salivary flow rate.

We did not stimulate salivary flow before the exposure to Coca-Cola^®^ to simulate the most unfavourable situation where a soft drink is consumed in the fasted state thus exhibiting its maximum erosive potential. The exposure to Coca-Cola^®^ in this study included holding 20 mL of the beverage in the mouth for 30 s, which is different from the way such beverages are consumed. This was performed for standardization purposes. The standard bolus size for a single swallowing in adults was found to be approximately 20–25 mL [68], while the oral transit time of liquids is about 1 s [69]. However, carbonated drinks are commonly held in the mouth for longer times (until all the bubbles have dissipated) [70]. Since the volume of a Coca-Cola^®^ can is 330 mL, which is approximately 15 sips, each 1–2 s long, the teeth are commonly exposed to the beverage for about 30 s.

Diet plays a key role in the development of enamel erosion [11,38]. Coca-Cola^®^ is one of the world’s most highly consumed commercial carbonated beverages [19] and has been shown to possess a high erosive potential [35,71,72]; therefore, in our study, we used rinsing with Coca-Cola^®^ to model a dietary acidic attack. A number of studies have shown that Coca-Cola^®^ decreased the salivary pH immediately after consumption [35,36,37,39,40], but generally it did not produce a decrease in salivary pH below 5.5 [35,36,37]. The pH value of 5.5 was found to be critical for enamel dissolution and caries development in vitro [56,61]; at the same time, in vivo this value varies over a wide range depending on the individual characteristics of the enamel and the content of mineral ions in plaque fluid and saliva [73,74,75]. In relation to erosion, critical pH is reversely proportional to the calcium and phosphate concentrations not only in the saliva and plaque fluid, but also in the foods and drinks. Therefore, it can be even lower if acidic products contain more calcium and phosphate than biofilm fluid [73,76,77,78]. In our study, the mean minimum pH after exposure to Coca-Cola^®^ was 6.3 ± 0.5, i.e., far above the theoretical critical level. These results are in accordance with those reported by previous studies [35,36,37].

The salivary clearance of an acidic drink is the time required for the salivary pH to reach the baseline level [38,79]. According to our results, the mean salivary clearance time of Coca-Cola^®^ was 27 min. In a study by Mojaver et al., the oral clearance rate of this beverage was found to be 30 min [71], while Barrajas-Torres et al. reported a gradual salivary pH recovery after the ingestion of Coca-Cola^®^ that took up to 45 min [36]. According to Tenuta et al., the salivary clearance time of Coca-Cola^®^ was 3 min [39]. Inconsistencies in the results of different studies may be explained by the differences in the ages, gender, and oral health status of the studies’ participants and differences in the studies’ designs.

Minimum salivary pH and salivary clearance may depend on drink-related factors (e.g., pH, titratable acidity, and composition [35,80]) and host-related factors (e.g., salivary buffer capacity and salivary flow rate [80]). In our study, the minimum salivary pH mainly correlated with salivary buffer capacity and salivation rate. Similar results were obtained by Tenouvo et al., who reported that the decrease in pH after consumption of the tested drinks was significantly less in subjects with a high salivary secretion rate compared to those with a low flow rate [35]. This may be explained by faster washing out of acids and the neutralizing effects of saliva buffering systems (mainly bicarbonates), which are activated with an increase in salivary secretion [37,80,81]. We found a positive correlation between salivary flow rate and buffer capacity. This finding corroborates those reported in the previous studies [82,83].

The neutralization of acids after soft drink consumption is a complex phenomenon, as salivary pH may also be influenced by organic acids produced by plaque microorganisms while metabolizing sucrose contained in these beverages [84,85]. Kumar et al. reported that the presence of plaque decreased the salivary pH at various time intervals between 0 and 60 min [86]. In our study, OHI-S plaque scores also correlated with salivary clearance of the acidic drink. The time required for acid neutralization was significantly longer in individuals with poorer oral hygiene. Interestingly, salivary clearance did not significantly correlate with salivary buffer capacity and salivation rate. This may be explained by the fact that salivary flow rate and buffer capacity mainly influence initial pH changes that occur within the first few minutes after the acidic attack, while further pH recovery may be hampered by acids produced by plaque microorganisms [27,87,88].

A number of studies have shown that the erosive potential of soft drinks may be reduced by increasing salivary flow rate [44,45,46], modification of beverages by adding calcium, phosphate, and fluoride [47,48,49], or using various oral care products immediately after the intake of soft drinks [57,60,61]. It has been shown that the use of various oral care products may result in rehardening of the enamel [60] and an increase in salivary pH [57] after an acidic attack. We found that rinsing with any one of the tested solutions resulted in a decrease in salivary clearance time and an increase in minimum pH, with no significant differences among the oral care products and tap water. Similarly, in a number of studies, water has been shown to be an effective measure to increase the salivary pH values [57,89] or even to increase enamel hardness [60] after the acidic challenge. This may be explained by the washing-out effect and eliminating acids from the oral cavity during rinsing.

On the other hand, when we plotted the pH curves, only dental foams and spray differed significantly from Coca-Cola^®^ in terms of the areas under the pH curves, while tap water did not. This could be due to the gustatory stimulation of the salivary flow by the tested foams and spray. A number of studies have suggested that salivation might be conditioned to sensory stimulation [90,91]. Additionally, the oral care products used in our study may provide beneficial effects in patients frequently consuming soft drinks due to their compositions and properties. BS is an alkaline spray with a pH of 8.8 which can directly neutralize acids. It has been shown that alkaline oral care products may increase salivary pH [57,58,59]. In a study by Dehgan et al., an alkaline component of a two-step mouthwash was used to reduce the erosive potential of an acidic drink [57]. They concluded that this mouthwash raised pH significantly higher than Listerine and water. WF contains 250 ppm fluoride. An increase in salivary pH has been reported in previous studies assessing the effect of various fluoride-containing oral care products [58,92,93]. Moreover, a considerable amount of the literature has reported a high potential of fluoride for surface remineralization after an acidic attack and protection from demineralization in an acidic environment [20,50,51,52]. The application of fluoride results in the formation of fluorohydroxyapatite layer on the tooth surface. This layer is less soluble and less susceptible to acid erosion [20,50,51,52]. BF active ingredients include Zn-hydroxyapatite and a probiotic. Remineralizing properties of hydroxyapatite are well documented [53,54,55,56,94,95]. Biomimetic (hydroxyapatite-containing) oral care products replicate the natural structure of teeth and can promote the remineralization of hard tooth tissues [53,54,55,56]. Particularly hydroxyapatite doped with Zn ions has been found to be an effective agent in mineralizing hard tooth tissues [94,95,96]. As it was mentioned above, BF also contains probiotics. There is some evidence that topical probiotics may increase salivary flow rate [97,98]. Also, probiotics have been reported to decrease plaque formation [99,100,101,102,103,104].

However, most of these effects require prolonged usage of the tested oral care products. In our study, we assessed only an immediate effect of the foams and spray, and further research of their long-term effects is needed. This can be considered a limitation of our study. Another limitation is that we did not assess mineralization and physical characteristics of hard tooth tissues after the exposure to the tested solutions. Finally, it is advisable for further studies to include patients from populations of different areas and ages, and to compare patients with enamel erosion with healthy ones.

## 5. Conclusions

Within the limitations of our study, we may conclude that rinsing with oral care products or tap water is an effective measure to increase the salivary pH values and salivary clearance rate after exposure to acidic beverage. Oral care products might provide additional benefits for decreasing the erosive potential of soft drinks, when the area under the pH curve is considered.

## Figures and Tables

**Table 1:** The characteristics of the tested solutions.

Short Name	Solution	Active Ingredient	pH	Titratable Acidity
Tap water	Tap water	Not applicable	pH = 7.3 ± 0.1	0.01 ± 0.0
Coca-Cola^®^	Coca-Cola^®^ (Coca-Cola Bottlers Georgia, Tbilisi, Georgia)		pH = 2.4 ± 0.1	2.3 ± 0.2
BF	Mousse Biorepair PERIBIOMA PRO (Coswell S.p.A., Funo di Argelato, BO, Italy)	Zinc hydroxyapatite and probiotics (*Lactobacillus*, *bifidobacterium*)	pH = 5.8 ± 0.1	0.03 ± 0.0
BS	BUCCOTHERM (Laboratoire ODOST, Castéra-Verduzan, France)	Castéra-Verduzan Thermal Spring water	pH = 7.8 ± 0.3	0.01 ± 0.0
WF	WATERDENT (LLC. Zelenaya dubrava, Dmitrov, Russia)	Olaflur	pH = 5.6 ± 0.2	0.26 ± 0.01

**Table 2:** Subject demographics.

Demographics	Values
Sex, *n* (%)	
Female	23 (72.9)
Male	9 (28.1)
Total	32 (100)
Age	
Mean (sd)	23.8 (6.1)
Median (Q1, Q3)	22 (21, 23)

(Q1; Q3)—interquartile range; sd—standard deviation.

**Table 3:** Oral health indicators.

Oral Health Indicators	Values
DMFT overall, teeth	
Mean (sd)	7.8 (4.8)
Median (Q1, Q3)	7.5 (5.0, 10.0)
DT, teeth	
Mean (sd)	1.3 (1.9)
Median (Q1, Q3)	0 (0, 3.0)
MT, teeth	
Mean (sd)	0.1 (0.3)
Median (Q1, Q3)	0 (0, 0)
FT, teeth	
Mean (sd)	6.2 (4.9)
Median (Q1, Q3)	6.0 (2.8, 8.3)
OHI-S, points	
Mean (sd)	1.0 (0.6)
Median (Q1, Q3)	1.0 (0.5, 1.3)
Plaque score, points	
Mean (sd)	0.9 (0.5)
Median (Q1, Q3)	0.9 (0.5, 1.2)
Calculus score, points	
Mean (sd)	0.1 (0.1)
Median (Q1, Q3)	0 (0, 0.2)
BI, points	
Mean (sd)	0.14 (0.15)
Median (Q1, Q3)	0.09 (0.03, 0.17)

(Q1; Q3)—interquartile range; sd—standard deviation.

**Table 4:** Salivary parameters.

Salivary Parameters	Values
Salivation rate, mL/min	
Mean (sd)	0.5 (0.2)
Median (Q1, Q3)	0.4 (0.35, 0.51)
Salivation rate, *n* (%)	
High	14 (43.75)
Medium	14 (43.75)
Low	4 (12.5)
Buffer capacity	
Mean (sd)	6.5 (0.8)
Median (Q1, Q3)	6.8 (6.0, 7.1)
Buffer capacity, *n* (%)	
High	30 (93.7)
Medium	2 (6.3)
Low	0 (0)

(Q1; Q3)—interquartile range; sd—standard deviation.

**Table 5:** The mean minimum pH, salivary clearance ^1^, and area under pH curve after exposure to Coca-Cola^®^ alone or that followed by the use of oral care products.

Tested Solutions	Min pH		Clearance ^1^		Area under Curve	
	Mean (sd)	Median (Q1; Q3)	Mean (sd)	Median (Q1; Q3)	Mean (sd)	Median (Q1; Q3)
Coca-Cola^®^	6.3 (0.5) ^a^	6.3 (5.9; 6.6)	27.3 (15.5) ^A^	30 (15; 40)	12.7 (11.0) ^a^	10.8 (3.5; 20.6)
Tap water	6.6 (0.5) ^b^	6.5 (6.3; 6.8)	17.3 (14.0) ^B^	15 (5; 21.3)	7.7 (9.4) ^ab^	5.0 (0.6; 11.0)
WaterDent	6.8 (0.6) ^b^	6.7 (6.4; 7.1)	17.2 (14.8) ^B^	12.5 (5; 25)	6.2 (8.3) ^b^	3.2 (0.4; 7.6)
Biorepair	6.7 (0.6) ^b^	6.8 (6.4; 7.0)	18.1 (15.0) ^B^	15 (5; 25)	5.8 (9.3) ^b^	1.6 (0.2; 7.9)
Buccotherm	6.7 (0.5) ^b^	6.8 (6.4; 7.1)	15.9 (15.3) ^B^	10 (5; 16.3)	7.3 (10.6) ^b^	2.2 (0.4; 10.7)
*p*-value ^2^	0.0000421		0.0225		0.000148	
Effect size ^3^	0.198 (small)		0.0890 (small)		0.177 (small)	

^1^ time required for salivary pH to reach baseline level; ^2^ according to Friedman’s test; ^3^ according to Kendall’s W; (Q1; Q3)—interquartile range; sd—standard deviation; A, B, a, b—different letters in a column indicate statistically significant differences between the studied solutions.

## Data Availability

The datasets used and/or analysed during the current study are available from the corresponding author upon reasonable request.
